# Interactive biomedical ontology matching

**DOI:** 10.1371/journal.pone.0215147

**Published:** 2019-04-17

**Authors:** Xingsi Xue, Zhi Hang, Zhengyi Tang

**Affiliations:** 1 College of Information Science and Engineering, Fujian University of Technology, Fuzhou, Fujian, China; 2 Intelligent Information Processing Research Center, Fujian University of Technology, Fuzhou, Fujian, China; 3 Fujian Provincial Key Laboratory of Big Data Mining and Applications, Fujian University of Technology, Fuzhou, Fujian, China; 4 Fujian Key Lab for Automotive Electronics and Electric Drive, Fujian University of Technology, Fuzhou, Fujian, China; 5 Key Laboratory of Hunan Province for Mobile Business Intelligence, Hunan University of Commerce, Changsha, China; Northeast Normal University, CHINA

## Abstract

Due to continuous evolution of biomedical data, biomedical ontologies are becoming larger and more complex, which leads to the existence of many overlapping information. To support semantic inter-operability between ontology-based biomedical systems, it is necessary to identify the correspondences between these information, which is commonly known as biomedical ontology matching. However, it is a challenge to match biomedical ontologies, which dues to: (1) biomedical ontologies often possess tens of thousands of entities, (2) biomedical terminologies are complex and ambiguous. To efficiently match biomedical ontologies, in this paper, an interactive biomedical ontology matching approach is proposed, which utilizes the Evolutionary Algorithm (EA) to implement the automatic matching process, and gets a user involved in the evolving process to improve the matching efficiency. In particular, we propose an Evolutionary Tabu Search (ETS) algorithm, which can improve EA’s performance by introducing the tabu search algorithm as a local search strategy into the evolving process. On this basis, we further make the ETS-based ontology matching technique cooperate with the user in a reasonable amount of time to efficiently create high quality alignments, and make use of EA’s survival of the fittest to eliminate the wrong correspondences brought by erroneous user validations. The experiment is conducted on the Anatomy track and Large Biomedic track that are provided by the Ontology Alignment Evaluation Initiative (OAEI), and the experimental results show that our approach is able to efficiently exploit the user intervention to improve its non-interactive version, and the performance of our approach outperforms the state-of-the-art semi-automatic ontology matching systems.

## Introduction

Ontologies have gained much importance in the past two decades, especially in the biomedical domain. Various biomedical ontologies such as Gene Ontology (GO) [1], National Cancer Institute (NCI) Thesaurus [2], Foundation Model of Anatomy (FMA) [3], and Systemized Nomenclature of Medicine (SNOMED-CT) [4] have emerged and been maintained, which have been widely used in the medical records annotation [5], medical data formats standardization [6], medical or clinical knowledge representation and integration [7], and medical decision making [8]. Due to continuous evolution of biomedical data, biomedical ontologies are becoming larger and more complex, which leads to the existence of many overlapping information. For example, NCI ontology defines the concept of “Myocardium” related to the concept “Cardiac Muscle Tissue” in FMA ontology, which describes the muscles surrounding the human heart. Since the utilization of these overlapping information is necessary for the integration, aggregation, and inter-operability among ontology-based biomedical systems, it is necessary to find the correspondences between these information, which is commonly known as biomedical ontology matching. However, matching biomedical ontologies is computationally intensive task with quadratic computational complexity [9], which arises from their characteristics: (1) biomedical ontologies often possess tens of thousands of classes, (2) biomedical terminologies are complex and ambiguous, frequently the same biomedical concept has several names, or the same terminology can be applied to two different entities. Although this challenge has attracted the interest of the community such as Ontology Alignment Evaluation Initiative (OAEI) which includes specific tracks on matching biomedical ontologies, the research on it is still in its infancy.

To efficiently match biomedical ontologies, it is critical to reduce the search space, which can improve the matching efficiency and the potential alignment’s quality. Recently, researchers have proposed various resolutions to reduce the search space, which mainly focus on the utilization of clustering and blocking strategies to reduce the search space [10–13]. Although divide-and-conquer strategy is a feasible solution for the large-scale ontology matching problem, it has two main issues: (1) the ontology partitioning algorithm cannot control the size of blocks, which may be too small or too large for matching, (2) the ontology partitioning process would make the elements on the boundaries of blocks lose some semantic information, which directly affect the quality of the alignment. Moreover, since none of the existing similarity measures can distinguish the biomedical concepts in all contexts, the user knowledge should be utilized in an automatic ontology matching process to ensure the quality of the final matching results [14]. To this end, a number of interactive ontology matching methods are developed, and various strategies on user interaction exploitation are presented. AgreementMakerLight (AML) [15] employs an interactive selection algorithm, which utilized the alignments returned by various ontology matchers to detect suspicious mappings. Above the threshold 70%, AML queries the user for suspicious mappings, otherwise, it rejects all the suspicious mappings. AML ensures that the reasonable workload for the user by setting the query limit as 45% of the determined correspondences for small scale ontology matching tasks, and 15% for the others. ALIN [16] generates an initial set of candidate correspondences, and requires the user to validate them. If the user judges a candidate mapping as correct, it will be moved to the final alignment. Then, ALIN removes all candidate mappings that are not consistent with the approved correspondences. The interactions continue until there are no more candidate correspondences left. LogMap [17] presents problematic mappings to the user for validation, and the validated results are utilized to detect the conflicts with already found mappings. LogMap allows to pause the user interaction and continue the validation work in the future. XMap [18] cooperates with the user in the post-matching steps to filter the final alignment. It uses two thresholds to implement this procedure, where the mappings with similarity value higher than the upper threshold are directly added to the final alignment, and those mappings with similarity values lower than the lower threshold are presented to the user for validation. The above interactive ontology matching systems exploit the user involvement in either pre-matching or post-matching phrase, and do not take the error made by a user into consideration, which can not ensure the quality of the ontology alignments.

Due to the complexity of the ontology matching problem (large-scale optimal problem with lots of local optimal solutions), Evolutionary Algorithm (EA) can present a good methodology for determining the ontology alignments [19]. The most notable one that utilizes EA to match ontologies is GOAL (Genetics for Ontology ALignments) [20], which determines the optimal weights to aggregate different alignments determined by various similarity measures. Alexandru et al. [21] further proposes to optimize both the aggregating weights and the threshold for filtering the final alignment to improve the alignment’s quality. GAOM (Genetic Algorithm based Ontology Matching) [22] tries to directly optimize the ontology alignment through the fitness function. However, the slow convergence and premature convergence are two main shortcomings of these EA-based matchers, which make them incapable of effectively searching the optimal solution for biomedical ontology matching problems. To improve the efficiency of EA-based matcher, in this paper, an Evolutionary Tabu Search (ETS) algorithm is proposed, which can improve EA’s performance by introducing the tabu search algorithm as a local search strategy into the evolving process. This marriage between global search and local search allows keeping high solution diversity via EA (reducing the possibility of the premature convergence) and increasing the convergence speed via the local search (improving the solution quality and thus makes the solutions approach to the optimal solution more quickly). On this basis, we further propose an interactive biomedical ontology matching technique, which can make the ETS-based ontology matching technique cooperate with a user in a reasonable amount of time to efficiently create high quality matchings, and makes use of EA’s survival of the fittest to eliminate the wrong correspondences brought by erroneous user validations. In particular, the contributions made in this paper are as follows:

- An interactive framework is proposed to match biomedical ontologies in an iterative way,
- An ETS-based ontology matching technique is presented to implement the efficient automatic matching process, which can adaptively determine the timing of getting a user involved,
- A hierarchy-based approach is presented, which can make use of partial biomedical concept mappings to reduce the algorithm’s search space.

The rest of the paper is organized as follows: Section 1 presents the framework of interactive biomedical ontology matching; Section 2 shows the automatic biomedical ontology matching technique based on ETS; Section 3 presents the interactivity during the evolving process of ETS; Section 4 presents the experimental studies and analysis; finally, Section 5 draws the conclusions and presents the future work.

## 1 Interactive biomedical ontology matching framework

**Fig 1:**
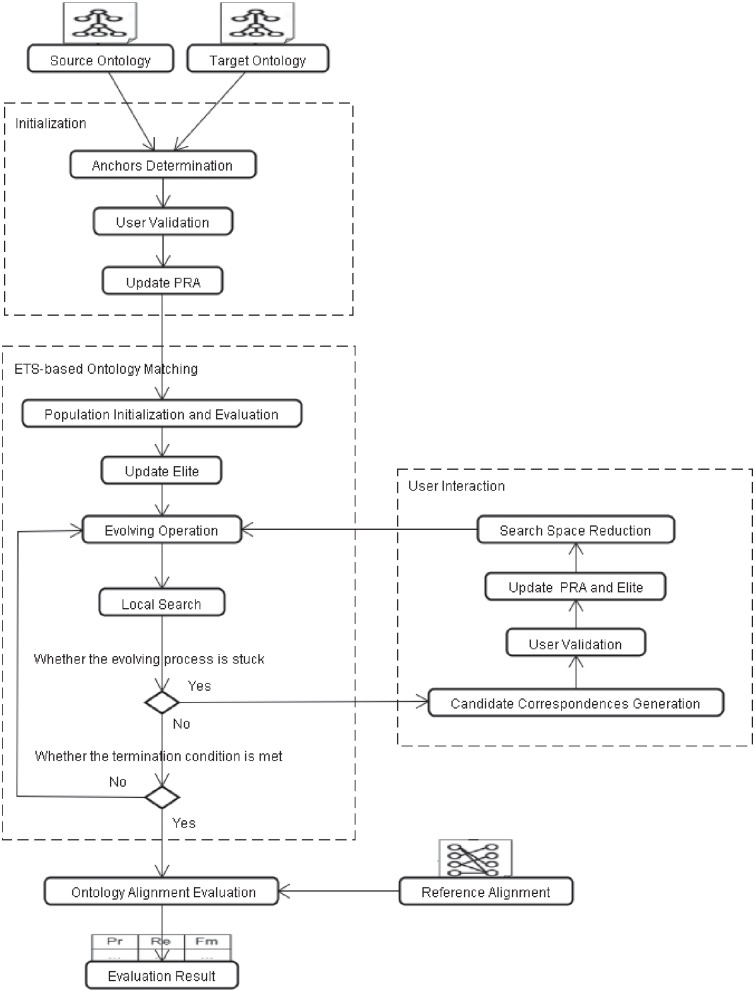
Interactive biomedical ontology matching framework.

In this work, the proposed interactive biomedical ontology matching framework is shown in Fig 1. As can be sen from the figure, three working phases, i.e. initialization, ETS-based ontology matching, and user interaction, are outlined by dotted-line boxes. A rectangle inside the dotted-line box represents a working step, and a rectangle with a picture outside the dotted-line box indicates the input or output data, e.g. source and target ontologies, reference alignment and evaluation result. Specifically, the description of three working phases is given as follows:

- Initialization: before matching biomedical ontologies, the anchors (high-confidence concept correspondences), are presented to a user for validation to initialize the Partial Reference Alignment (PRA),
- ETS-based matching process: ETS algorithm is utilized to match the biomedical ontologies in an iterative way, and when the evolving process gets stuck, the algorithm will get a user involved,
- User interaction: the candidate correspondences are presented to a user for validation, and the validated results are further used to update PRA, elite, and reduce the search space of ETS through the hierarchy-based approach.

## 2 Evolutionary tabu search algorithm based biomedical ontology matching

### 2.1 Biomedical ontology matching problem

A biomedical ontology *O* can be defined as a 5-tuple (*C*, *P*, *I*, *A*), where *C*, *P*, *I*, *A* are referred to the set of classes, properties, instances and axioms, respectively. In general, class, property and instance are referred to as entities. A correspondence can be defined as a 3-tuple (*e*_1_, *e*_2_, *n*), where *e*_1_ and *e*_2_ are the entities from two ontologies, *n* ∈ [0, 1] is the similarity value between *e*_1_ and *e*_2_. The correspondence set is called an ontology alignment *A*, and PRA is a set of correct correspondences that are provided by a domain expert [23]. Given a partial reference alignment *PRA*, a partial alignment *A*_*p*_ is the subset of *A* which contains all elements in *A* and shares at least one class with an element in *PRA* [23]:

$$\begin{array}{c} A_{p}=\left\{\left(e_{1},e_{2},n,=\right)\in A|\exists e_{1}^{\prime },n^{\prime }:\left(e_{1}^{\prime },e_{2},n^{\prime },=\right)\in PRA\right\}\cup \left\{\left(e_{1},e_{2},n,=\right)\in A|\exists e_{2}^{\prime },n^{\prime }:\left(e_{1},e_{2}^{\prime },n^{\prime },=\right)\in PRA\right\} \end{array}$$ (1)

Given an alignment *A*′, whose recall, precision and f-measure on PRA [24] are defined as follows:

$$\begin{array}{c} precision_{pra}=\frac{|A^{\prime }\cap A|}{\left|A\right|} \end{array}$$ (2)

$$\begin{array}{c} recall_{pra}=\frac{|A^{\prime }\cap A|}{|A^{\prime }|} \end{array}$$ (3)

$$\begin{array}{c} f-measure_{pra}=2\times \frac{precision\times recall}{precision+recall} \end{array}$$ (4)

On this basis, the optimal model of biomedical ontology matching problem is defined as follows:

$$\left\{ \begin{array}{c} max\;f-measure_{pra}\left(X\right)\\ s.t.\;X={\left(x_{1},x_{2},\cdots ,x_{|O_{1}|}\right)}^{T}\\ \;\;\;x_{i}\in \left\{1,2,\cdots ,\right|O_{2}\left|\},i=1,2,\cdots ,\right|O_{1}| \end{array}\right.$$ (5)

where |*O*_1_| and |*O*_2_| refer to the cardinalities of two biomedical ontologies *O*_1_ and *O*_2_ respectively, *x*_*i*_, *i* = 1, 2, ⋯, |*O*_1_| is the *i*-th correspondence.

In the next, the ETS algorithm is presented in details to solve this problem and implement the automatic ontology matching process.

### 2.2 Partial reference alignment initialization

In this work, we utilize the HashMap http://en.wikipedia.org/wiki/Hash_table to determine the anchors, i.e. the entities with identical labels. In particular, firstly, each class of source (or target) ontology is stored in the source (or target) HashMap as the key and its label is the value associated with the key. Then, the values of source HashMap are used to query the target HashMap to determine the highly similar mappings, whose time complexity is *O*(*n*). Finally, a user is asked to validate the anchors, and those are judged as true will be further utilized to construct the PRA.

### 2.3 Evolutionary tabu search algorithm

Since modeling the biomedical ontology matching problem is a complex (nonlinear problem with many local optimal solutions) and time-consuming task (large scale problem), particularly when the number of biomedical concepts is significantly large, EA can represent an efficient approach for addressing it. However, the slow convergence and premature convergence are two main shortcomings that make EA-based ontology matcher incapable of effectively searching the optimal solution for biomedical ontology matching problem. Starting from these considerations, this work proposes an ETS algorithm which combines EA (global search) and tabu search algorithm (local search) to implement the automatic searching process, which can keep high population diversity and increase the convergence speed via the local search. For the sake of clarity, the pseudo-code of ETS algorithm is presented as follows:

**Algorithm 1** Evolutionary Tabu Search Algorithm

Initialize the Generation *t* = 0;

Initialize the Population *P*_*t*_;

Evaluate(*P*_*t*_);

**while** *t* < *MaxGeneration* **do**

 $P_{t_{\prime }}^{\prime }=\text{Crossover}\left(P_{t}\right);$

 $P_{t}^{\prime \prime }=\text{Mutation}(P_{t}^{\prime });$

 Evaluate($P_{t}^{{}^{\prime \prime }}$);

 localSearch();

 saveElite();

 $P_{t}=\text{Select}\left(P_{t}^{\prime \prime }\right);$

 *t* = *t* + 1;

**end while**

In the next, three key components of ETS algorithm are presented in details, i.e. encoding mechanism, genetic operator and local search process.

#### 2.3.1 Encoding mechanism

Let |*C*_1_| and |*C*_2_| be the cardinalities of the source concept set *C*_1_ and target concept set *C*_2_, respectively. Each chromosome in the population would be a one-dimensional array with |*C*_1_| elements, and the elements are denoted as: *N*_1_ *N*_2_⋯*N*_|*C*_1_|_, where *N*_*i*_ ∈ *ω*_*i*_ = {0, 1, ⋯, |*C*_2_|}, which means the *i*th concept in *C*_1_ is mapped to the *N*_*i*_th concept in *C*_2_. In particular, when *N*_*i*_ = 0, the *i*th concept is not mapped to any concept in *C*_2_.

#### 2.3.2 Genetic operators

In this work, we evaluate the population by *f* − *measure*_*pra*_ and then use a roulette wheel selection method, where an individual is given a probability of being selected that is directly proportionate to its fitness value, and in this way, the most suitable individuals will have more opportunities of reproduction, while the less suitable individuals also have the chance of reproduction. After choosing two individuals (the parents), we use the one-cut-point crossover operator to produce their offsprings: first, a cut position in two parents is randomly determined and this position is a cut point which cuts each parent into two parts: the left part and the right part; then, the right parts of them are switched to form two children. With respect to the mutation operator, for each gene bit *N*_*i*_, we check if the mutation could be applied according to the mutation probability, and if it is, the value of *N*_*i*_ is then randomly changed to a value in its corresponding search space *ω*_*i*_.

#### 2.3.3 Local search process

A local search process performs iterative search for the optimal solution in the neighborhood of a candidate. In order to tradeoff between the local search and the global search, the local search process in our work is designed according to the following rules:

- the local search is applied within each evolutionary cycle,
- the local search is executed after crossover and mutation,
- the local search is applied to the best individual of population,
- the local search method is the tabu search algorithm.

Tabu search concerns with imposing restrictions to guide a search process to negotiate otherwise difficult regions, where the restrictions can operate by direct exclusion of search alternatives classed as “forbidden”. The implementation of tabu search uses an array to describe the visited solutions, and if a potential solution has been previously visited within a certain short-term period, it is marked as “tabu” (forbidden) so that the algorithm does not consider that possibility repeatedly. Given a tabu matrix *TM* = [*TV*_1_, *TV*_2_, ⋯, *TV*_|*C*_1_|_] where the *i*th tabu list *TV*_*i*_ = (*tv*_1_, *tv*_2_, ⋯, *tv*_*tLength*_)^*T*^, *i* = 1, 2, ⋯, |*C*_1_|, *tv*_*j*_ ∈ 0, 1, 2, ⋯, |*C*_2_|, the pesudo-code of tabu search algorithm is given as follows:

**Algorithm 2** Local Search Process

*iterNum* = 0;

**while** *iterNum* < *maxIterNum* **do**

 **for** *n* = 0;*n* < *neighborScale*;*n* + + **do**

  *solution*_*new*_ = *solution*_*elite*_.*copy*();

  **for** *i* = 0;*i* < *solution*_*new*_.*length*;*i* + + **do**

   **if** *random*(0, 1) < *mutationProbability*_*LS*_ **then**

    $solution_{new}[i]=random(\{0,1,\cdots ,|C_{2}\left|\right\}-\left\{tv_{j}^{i}\in TV_{i}\right\})$;

   **end if**

  **end for**

  append *solution*_*new*_[*i*] to *neiborhood*;

 **end for**

 *solution*_*localElite*_ = elite in *neiborhood*;

 *compete*(*solution*_*Elite*_, *solution*_*localElite*_);

 **if** *winner* == *solution*_*localElite*_ **then**

  **for** each *TV*_*i*_ ∈ *TM* **do**

   **if** *TV*_*i*_ is not full **then**

    append *solution*_*localElite*_[*i*] to *TV*_*i*_;

   **else**

    replace $tv_{j}^{i}\in TV_{i}$, whose corresponding class in *C*_2_ has the lowest similarity value with *c*_*i*_, with *solution*_*localElite*_[*i*];

   **end if**

  **end for**

  *iterNum* ++;

 **else**

  break;

 **end if**

**end while**

During the evolving process, if *solution*_*elite*_ keep unchanged for certain generations, each $tv_{j}^{i}\in TV_{i}$, whose corresponding class in *C*_2_ has the highest similarity value with *c*_*i*_, will be removed.

## 3 User interaction

Since matching biomedical ontology matching is a complex task, ETS-based matching results need to be validated by a user to ensure the alignment’s quality and improve the algorithm’s efficiency [25]. However, it is impractical to require a user to validate all the correspondences at a time, which is both time-consuming and error prone. Thus, how to reduce a user’s workload is the first question we need to answer when implementing an effective user interaction. In addition, how to effectively exploiting the limited user intervention to improve the matching process’s efficiency is the second question that we need to answer. In this work, we get a user involved only when ETS gets stuck, and present the most problematic correspondences (those with low similarity measure value) to him for validation to reduce his workload. When a user validates all the correspondences, the validated results will be further utilized to reduce each gene bit’s search space through a hierarchy-based approach, which can improve the efficiency of hereafter matching process.

### 3.1 Biomedical concept similarity measure

Similarity measure is a function that takes as input two concepts and outputs a score between 0, which means two concepts are completely different, and 1, which means two concepts are identical. In particular, we first construct a profile for each biomedical concept by collecting the label, comment, and property labels from itself, and all its direct descendants. Then the similarity value between two biomedical class *c*_1_ and *c*_2_ is measured by calculating the similarity of their corresponding profiles *P*_1_ and *P*_2_, which is defined in Eq 6.

$$\begin{array}{c} sim\left(c_{1},c_{2}\right)=\frac{\sum_{i=1}^{|P_{1}|}\max_{j=1\cdots |P_{2}|}\left(sim^{\prime }\left(p_{1i},p_{2j}\right)\right)+\sum_{j=1}^{|P_{2}|}\max_{i=1\cdots |P_{1}|}\left(sim^{\prime }\left(p_{1i},p_{2j}\right)\right)}{|P_{1}\left|+\right|P_{2}|} \end{array}$$ (6)

where:

- |*P*_1_| is the number of elements of *P*_1_ and |*P*_2_| is the number of elements of *P*_2_,
- *p*_1*i*_ is the *i*th property of *P*_1_ and *p*_2*j*_ is the *j*th property of *P*_2_, e.g. the label or comment in concept description profile,

Here, *sim*′(*p*_1*i*_, *p*_2*j*_) calculates the similarity value of two profile elements by N-gram distance [26], which is the most performing string-based similarity measure for the biological ontology matching problem, and a linguistic measure, which calculate a synonymy-based distance through Unified Medical Language System (UMLS) [27]. To be specific, given two words *w*_1_ and *w*_2_, their similarity *sim*(*w*_1_, *w*_2_) is equal to 1 when two words are synonymous, and otherwise, *N* − *gram*(*w*_1_, *w*_2_).

### 3.2 Improve the efficiency of matching process

It is the large search space that makes EA-based ontology matcher difficult to match the biomedical ontologies, thus, how to reduce the search space is critical for a biomedical ontology matching technique. In this work, we propose a hierarchy-based approach to exploit the validated results to effectively reduce the ETS algorithm’s search space. Our proposal works on the basis of two observations [28]: (1) a biomedical ontology is often composed of the hierarchies organized by “is-a” relationship, and a correct alignment should be consistent with such hierarchies, (2) an alignment between two biomedical ontologies has locality, i.e. most class of a region in one ontology will match to the classes of a region in another ontology, and the search space reducing process is as follows:

- if a user judges a source concepts *c*_*i*_ and a target *c*_*j*_ are identical, the sub-concepts(or super-concepts) of *c*_*i*_ and super-concepts(or sub-concepts) of *c*_*j*_ should not match, i.e. *c*_*j*_’s super-concepts’ indexes will be removed from the search space *ω*′ of each *c*_*i*_’s sub-concept $c_{i}^{\prime }$’s corresponding gene bit, and *c*_*j*_’s sub-concepts’ indexes will be removed from the search space *ω*″ of each *c*_*i*_’s super-concept $c_{i}^{\prime \prime }$’s corresponding gene bit,
- if a user judge a source concepts *c*_*i*_ and a target *c*_*j*_ are not the same, the neighborhood of *c*_*i*_ do not match *c*_*j*_ too, i.e. *c*_*j*_’s index will be removed from the search space *ω*‴ of each *c*_*i*_’s neighbor $c_{i}^{\prime \prime \prime }$’s corresponding gene bit. In particular, *c*_*i*_’s neighborhood include *c*_*i*_’s direct super-concept, sub-concept and siblings.

By omitting dissimilar correspondences, the search space of ETS algorithm can be significantly reduced after each user interaction, as well as the alignment’s quality potentially.

## 4 Experimental studies and analysis

In this work, we exploit the Anatomy http://oaei.ontologymatching.org/2016/anatomy/index.html and Large Biomed http://www.cs.ox.ac.uk/isg/projects/SEALS/oaei/2016/ track to study the effectiveness of our approach, which are provided by OAEI 2016 http://oaei.ontologymatching.org/2016. The experiment allows the matching approaches to ask an oracle who will then tell the matcher whether the correspondence is right or wrong. Tables 1, 2 and 3 show the mean value of f-measure of the alignments obtained by our approach in thirty independent runs and the results obtained by the participants of OAEI. The symbols *r*, *p* and *f* in the tables stand for recall, precision and f-measure, respectively, and $\bar{f}$, $\bar{r}$ and $\bar{p}$ respectively stand for the matcher’s non-interactive version’s f-measure, recall and precision. In this experiment, we use three metrics, i.e. f-measure, runtime and the mean improvement per request, to evaluate the performances of the interactive biomedical ontology matchers. In particular, f-measure and runtime can be used to measure the effectiveness of semi-automatic ontology matching technique, and the mean improvement per request can measure the efficiency of the user involvement.

**Table 1:** Comparison between OAEI 2016’s participants and our approach on interactive anatomy track.

	ALin	AML	LogMap	XMap	Our Approach
$\bar{f}(\bar{r},\bar{p})$	0.50 (0.33, 0.98)	0.94 (0.93, 0.95)	0.87 (0.84, 0.91)	0.89 (0.86, 0.92)	0.82 (0.80, 0.84)
*f* (*r*, *p*)	0.85 (0.74, 0.99)	0.95 (0.94, 0.96)	0.90 (0.84, 0.98)	0.89 (0.86, 0.92)	0.97 (0.96,0.97)
Total Requests	803	241	590	35	262
Mean Improvement per Request	4.35 × 10^−4^	4.14 × 10^−5^	5.08 × 10^−5^	0	5.72 × 10^−4^
Runtime (sec)	505	48	27	49	23

**Table 2:** Comparison between OAEI 2016’s participants and our approach on the interactive Large Biomedic track: FMA-NCI.

	Alin	AML	LogMap	XMap	Our Approach
$\bar{f}(\bar{r},\bar{p})$	0.62 (0.45, 0.99)	0.93 (0.90, 0.96)	0.92 (0.89, 0.94)	0.93 (0.90, 0.97)	0.92 (0.90, 0.93)
*f* (*r*, *p*)	0.77 (0.63, 0.99)	0.95 (0.91, 0.99)	0.94 (0.90, 0.99)	0.94 (0.90, 0.99)	0.97 (0.97, 0.98)
Total Requests	653	449	1131	188	289
Mean Improvement per Request	2.29 × 10^−4^	4.54 × 10^−5^	1.76 × 10^−5^	5.31 × 10^−5^	5.19 × 10^−4^
Runtime (sec)	5895	60	38	50	32

**Table 3:** Comparison between OAEI 2016’s participants and our approach on the interactive Large Biomedic track: SNOMED-NCI.

	AML	LogMap	XMap	Our Approach
$\bar{f}(\bar{r},\bar{p})$	0.79 (0.71, 0.90)	0.77 (0.66, 0.92)	0.69 (0.56, 0.91)	0.77 (0.72, 0.82)
*f* (*r*, *p*)	0.83 (0.72, 0.97)	0.79 (0.66, 0.98)	0.72 (0.59, 0.92)	0.86 (0.86, 0.87)
Total Requests	2730	5596	11689	1620
Mean Improvement per Request	1.46 × 10^−5^	3.57 × 10^−6^	2.56 × 10^−6^	2.60 × 10^−5^
Runtime (sec)	730	628	984	492

The configuration of EA in our work follows the following principles:

- In our work, since the EA works mainly based on the crossover operator and is aided by the mutation operator, the crossover possibility should be larger and the mutation possibility just the opposite. However, if the value of the crossover operator is too great, excess solutions would appear which might increase the cost of computation. Therefore, the suggested range of crossover probability is [0.2, 1], and through the preliminary experiment, we find that the results obtained with the crossover probability 0.85 and the mutation probability 0.02 are acceptable for various heterogeneous problem in all testing cases.
- Since the local searching process requires producing a local searching population with high diversity, the mutation possibility of local search should be higher than that of the genetic algorithm. However, if the value is too large, the produced individual might not be the “neighbor” of the local searching target. Therefore, the suggested range of mutation probability is [0.2, 0.8], and through the preliminary experiment, we find that the mutation probability 0.5 works better.
- The population size, local Search intensity and maximum number of generation for termination depend on the scale of the problem, the suggested ranges for them are [50, 120], [10, 40] and [1500, 3500], respectively. Since the problem scale in our work is relatively large, we set the size of population, local Search intensity and the maximum number of generation as 100, 30 and 3000 respectively.

In our work, **we use the following parameters** which represent a trade-off setting obtained in an empirical way to achieve the highest average alignment quality on all testing cases of exploited dataset. Through the configuration of parameters chosen in this way, it has been justified by the experiments that parameters chosen are robust for all the heterogeneous problems presented in the benchmarks, and it is hopeful to be robust for the common heterogeneous situations in the real world.

- Numerical accuracy = 0.01,
- Population size = 100,
- Crossover probability = 0.85,
- Mutation probability = 0.02,
- Local search’s mutation probability = 0.5,
- Local Search intensity = 30 iterations,
- Maximum number of generation = 3000,

In addition, in order to compare with the participants of OAEI 2016, we run our approach on Conference and Anatomy tracks on a server with Intel Xeon E5-2643 CPU @ 3.46 GHz x 6 cores and 8GB RAM, and Large Bio track and Phenotype track on a laptop with an Intel Core i7-4600U CPU @ 2.10GHz x 4 and allocated 15GB RAM. The operating system of both machines is Linux.

### 4.1 Anatomy track

The anatomy track is a large ontology matching task about matching the Adult mouse anatomy (2744 classes) and a part of the NCI Thesaurus (3304 classes) which describes the human anatomy. Adult mouse anatomy is a structured controlled vocabulary describing the anatomical structure of the adult mouse, whereas NCI depicts the human anatomy for the purpose of cancer research.

As can be seen from Table 1, our approach’s f-measure is the highest. In particular, comparing with the non-interactive version of our approach, both recall and precision are improved by 20% and 15% respectively, which shows that our approach can effectively exploit the user intervention to improve the alignment quality. In addition, because of the high efficiency brought by the hierarchy-based approach, our approach only takes 23 seconds to obtain the ontology alignment, which is the lowest among all matching systems. Our approach’s mean improvements per request are all higher than other systems, which illustrate that our approach can efficiently utilize the user involvement’s value. With the introduction of an erroneous oracle and moving towards higher error rates, each system’s performance starts to deteriorate in comparison to the all-knowing oracle. To sum up, our approach can efficiently exploit the user involvement to achieve the great improvement.

### 4.2 Large Biomedic track

This track aims at finding alignments between the large and semantically rich biomedical ontologies FMA, SNOMED, and NCI, which contains 78,989, 306,591 and 66,724 classes, respectively.

On the first track of Large Bio, as can be seen from Table 2, our approach improves the non-interactive version by 5.43% in terms of f-measure, comparing with Alin’s 2.4%, AML’s 2.15% and LogMap’s 2.17% and XMap’s 1.0%. Therefore, our approach’s user validation exploitation is effective, which makes our approach can efficiently deal with the large scale ontology matching problem and improve the ontology alignment’s quality. Since our approach can effectively reduce the number of user interaction and exploit the user validation’s value, the mean improvement per request of our approach is much higher than other systems. Last but not least, due to the efficiency brought by the search space reducing approach, the average runtime of our approach also is less than other systems.

As shown in Table 3, our approach obtains the highest f-measure. Comparing with the non-interactive version, our approach’s recall and precision are both improved by 19.44% and 6.09% respectively, which shows that our approach can effectively utilize the user intervention to improve the alignment quality. In addition, the mean improvement per request of our approach is also higher than other systems, but the mean runtime is the lowest under all the user error rates. In addition, our approach’s mean improvements per request are higher than other systems. To sum up, our approach is able to efficiently exploit the user involvement to obtain high quality ontology alignments when solving large scale biomedical ontology matching problem.

To conclude, through the comparison with OAEI’s participants in the interactive ontology matching tracks with different scales, our approach is able to more effectively exploit the user validation to improve the performance of its non-interactive version, and the qualities of the alignments obtained by our approach with three user error rates ranging from 0.1 to 0.3 are all better than the state-of-the-art interactive biomedical ontology matching techniques.

## 5 Conclusion and future work

To efficiently match biomedical ontologies, in this work, an interactive biomedical ontology matching approach is proposed, which can effectively utilize the user’s knowledge to guide the ETS-based ontology matcher’s search direction and improve its efficiency by reducing the algorithm’s search space. The experimental results show that our approach is able to efficiently exploit the user validation to improve its non-interactive version, and the performance of it outperforms the state-of-the-art interactive biomedical ontology matching techniques. In the future, we are interested in the strategies that can reuse a user’s validation results to further reduce the search space of the algorithm. In addition, we are also interested in decreasing the user’s error rate by warning him when contradicting validations are made.
